# Supplementary material for: MethylScore, a pipeline for accurate and context-aware identification of differentially methylated regions from population-scale plant whole-genome bisulfite sequencing data
Source: Quant Plant Biol. 2022 Sep 26;3:e19. doi: 10.1017/qpb.2022.14 (PMC10095865; doi:10.1017/qpb.2022.14)
Supplement: Supplementary file 1 [file S2632882822000145sup001.docx]

### Supplemental Material to:

### MethylScore, a pipeline for accurate and context-aware identification of differentially methylated regions from population-scale plant WGBS data

## *Patrick Hüther, Jörg Hagmann, Adam Nunn, Rahul Pisupati, Ioanna Kakoulidou, David Langenberger, Detlef Weigel, Frank Johannes, Sebastian J. Schultheiss, & Claude Becker*

This supplemental material file contains:

- Supplementary Methods
- Supplementary Figures 1-9
- Supplementary References

## Supplementary Methods

##### Pipeline strategy

To start the pipeline (Supplementary Figure 1), users need to provide a two-column sample sheet with sample identifiers and file paths to the respective *bam* or *bedGraph* files as well as to the reference genome sequence in *fasta* format. If multiple lines in the sample sheet share the same sample identifier, MethylScore will assume technical replication and merge the files prior to further processing.

First, the *samtools sort* [[1]](https://paperpile.com/c/xAKEZR/artBC) function sorts the alignments by genomic coordinates. As many library preparation protocols for bisulfite sequencing incorporate a PCR amplification step during the protocol, alignments can be optionally processed with *picard MarkDuplicates* [*[2]*](https://paperpile.com/c/xAKEZR/zOhN4) to avoid potential bias by non-uniform read amplification. For the next steps up to the methylated region calling, the alignments are split by chromosome or contig, facilitating full parallelization of downstream processing, which is open to user configuration in order to suit the available computational resources. *MethylDackel extract* (*https://github.com/dpryan79/MethylDackel*) is used for tabulation of single-cytosine information per sample.

The consensus files for each sample and chromosome are merged into a uniform file format containing methylation information for all cytosines across all samples, together with information about sequence context and strand information for each cytosine. After merging individual chromosomes, the resulting genome matrix file, a single file containing all of the above information for every sample and for each cytosine covered in at least one sample, is compressed with *bgzip* and indexed with *tabix*, allowing for targeted queries of regions from the compressed file.

The genome matrix serves as the input for iterative training of one Hidden Markov Model (HMM) per sample. It is implemented as a two-state (unmethylated/methylated) model, designed to learn three context-specific methylation rate distributions by fitting distinct beta-binomial distributions, thus reflecting the underlying biologically distinct control of these sequence contexts in plants. The two-state HMM is trained iteratively using the Baum-Welch algorithm for each of the samples as described by [[3]](https://paperpile.com/c/xAKEZR/6IuHF) with two key adaptations. First, to account for the fact that, in contrast to DNA methylation in mammals, plant genomes often only harbor DNA methylation in a small fraction of the genome, the original implementation was adapted to detect *hyper*-methylated rather than *hypo*-methylated regions, effectively achieved by inverting the methylation rates. Second, we adapted the extension to a total of three sequence contexts, allowing the model to learn distinct parameters independently for CG, CHG and CHH sequence contexts for both states. This results in a total of six distributions with corresponding emission probabilities as well as transition probabilities between the methylated and unmethylated states.

Based on the resulting parameterization of the trained model, the genome space is segmented into methylated and unmethylated regions by probabilistic classification, yielding genomic coordinates of MRs in *bed* format. These regions are split into batches to facilitate the parallel processing of the ensuing statistical testing strategy. Using an iterative *k*-means algorithm to determine the most suitable number of groups [[4]](https://paperpile.com/c/xAKEZR/hw7vk), samples are clustered according to their mean methylation rates within candidate DMRs. Clusters for each sequence context are assessed for statistical significance by employing log-likelihood ratios based on observed read counts under the beta-binomial assumption and testing against a chi-squared distribution with six degrees of freedom as outlined in [[5]](https://paperpile.com/c/xAKEZR/CzcUP), followed by FDR control using Storey’s method [[6]](https://paperpile.com/c/xAKEZR/fq56L).

Given the parameter estimates after model convergence for a maximum of 30 iterations, the most likely path is determined by Posterior Decoding and used for probabilistic segmentation of the genome into methylated regions (MRs). During this step, MR breakpoints are introduced at chromosome boundaries, and in regions lacking methylation information in intervals of at least 100 bp in length (parameter “DESERT_SIZE”). This user-defined parameter prevents extending MRs over stretches of missing data that completely lack methylation information. Having segmented the genome into MRs, MethylScore then applies two complementary population-scale approaches to reduce complexity of the statistical testing framework. First, candidate regions for significance testing are restricted to those displaying a 20% change in MR frequency (parameter “MR_FREQ_CHANGE”) across samples, indicative of natural region boundaries that are widespread in the sample population (Supplementary Figure 2). For calling less frequent methylation changes in the population, the MR frequency parameter can be adjusted to lower values. Other default parameters have been devised for broad use to filter out (D)MRs with untypically low methylation support for typical BS-Seq experiments of today, requiring low minimum numbers of covered cytosines in (D)MRs (5 in DMRs, 20 in MRs; parameters “MR_MIN_C”/”DMR_MIN_C”) with a minimum coverage (3x in DMRs, 1x in MRs; parameters “MR_MIN_COV”/“DMR_MIN_COV”). The second approach used by MethylScore to further decrease the number of statistical tests, is to employ *k*-means clustering in order to group samples by mean methylation rates within any given MR. For each candidate region, cluster centers are searched to minimize the within-group variance. The value of *k* is iteratively incremented starting from *k* = 2, until the pairwise comparison of all cluster centers results in a methylation difference of less than 20 percentage points (parameter “CLUSTER_MIN_METH_DIFF”), in which case the previous *k* is chosen. This parameter lets the user choose a target minimum methylation difference between groups of samples and thus can be effectively used to discard likely biologically irrelevant DMRs with only a few percentage points methylation difference. At the same time, the ability to identify DMRs occuring in single samples is retained, provided the methylation difference between remaining cluster centers exceeds the predefined value. Although this procedure depends on many parameters, we expect that most default values will be reasonable for different species and data sets. Methylated regions are called unsupervised with a model that trains on a species’ characteristic methylation pattern from the data. The two most influential parameters of MethylScore, “MR_FREQ_CHANGE” and “CLUSTER_MIN_METH_DIFF”, offer the possibility to filter out candidate DMRs upfront that are likely being discarded after statistical testing according to the user’s research question. If methylation changes private to very few samples should be retained, “MR_FREQ_CHANGE” should be set low (the lowest meaningful value is 1/*n* where *n* is the number of biological samples). If only slight methylation changes and thus lowly discriminatory DMRs should be retained, “CLUSTER_MIN_METH_DIFF” should be reduced from 20% to a lower value.

In a last step, DMRs that pass the FDR-corrected significance threshold are merged, yielding one *bed* file per methylation context containing DMRs between sample clusters, assuming that observed read counts follow beta-binomial distributions. For each pair of clusters, a total of three distributions are approximated from observed read counts within candidate region boundaries: one for each cluster segment and a joint distribution obtained from the two merged segments. Log-odds ratios are then computed between joint and cluster-specific estimates and tested against a chi-squared distribution with six degrees of freedom. Resulting p-values are subsequently corrected for multiple tests as described in [[7]](https://paperpile.com/c/xAKEZR/AZd3h).

##### Implementation in Nextflow

To achieve high standards of reproducibility and portability of pipeline execution across different compute infrastructures, the workflow manager Nextflow [[8]](https://paperpile.com/c/xAKEZR/ogr06) was chosen to implement MethylScore. Nextflow enables containerized execution using a prebuilt Docker image (available at https://quay.io/repository/beckerlab/methylscore), that contains all necessary dependencies including *perl* libraries, the pre-compiled HMM implementation, and version-pinned *bioconda* packages. Furthermore, Nextflow ensures efficient use of available computational infrastructure by straightforward parallelization of tasks, following a scatter-gather strategy. Time and memory requirements for individual pipeline steps are shown in Supplementary Figure 8.

##### Re-analysis of published datasets

WGBS reads were retrieved from the ENA and GEO repositories PRJEB26932 [[9]](https://paperpile.com/c/xAKEZR/uey7n), PRJEB12413 [[10]](https://paperpile.com/c/xAKEZR/niFZ2), GSE137754 [[11]](https://paperpile.com/c/xAKEZR/TUbmE) GSE42410 [[12]](https://paperpile.com/c/xAKEZR/jV4Wq) and [GSE171414](https://www.ncbi.nlm.nih.gov/geo/query/acc.cgi?acc=GSE171414) [[13]](https://paperpile.com/c/xAKEZR/C2opY)

Raw data were trimmed and mapped to the TAIR10 [[14]](https://paperpile.com/c/xAKEZR/RUBMW) reference genome for *A. thaliana* data and the IGRSP-1.0 genome [[15]](https://paperpile.com/c/xAKEZR/961Aa) for *O. sativa* spp. *japonica* data using v1.6 of the *nf-core methylseq* pipeline [[16]](https://paperpile.com/c/xAKEZR/3LHRw). Standard settings for Illumina adapter trimming, and the default aligner *bismark* [[17]](https://paperpile.com/c/xAKEZR/LTz5u) were used with addition of the --relax_mismatches preset. The rich quality control output of the pipeline was used to assess methylation bias on both read ends and affected bases were ignored for downstream analysis with MethylScore.

##### 1001 Methylomes analysis

Single-cytosine metrics obtained from the *A. thaliana* 1001 Methylomes dataset [[18]](https://paperpile.com/c/xAKEZR/KVnQV) were used to call DMRs in pairwise comparisons between 645 accessions using Col-0 as the point of reference. Overlapping regions found in multiple comparisons were merged using the *plyranges* (v.1.14.0 [[19]](https://paperpile.com/c/xAKEZR/fSic8) *reduce_ranges()* function in *R* (v4.1.2 [[20]](https://paperpile.com/c/xAKEZR/lORcQ)) For each sequence context, methylation rates within all identified DMRs were retrieved and used to seek associations between the average methylation rate for each accession within each DMR and the underlying genotype by conducting Genome-Wide Association Studies (GWAS) using the *Limix* framework [[21]](https://paperpile.com/c/xAKEZR/gaLxL) for linear mixed models using an in-house python implementation packaged into a containerized Nextflow pipeline (https://gitlab.lrz.de/beckerlab/gwas-nf).

In brief, our GWAS pipeline retrieves the average methylation rate for each accession within a given DMR and estimates population structure from all SNPs using the *limix.stats.linear_kinship* function in *Limix*. GWAS is performed via the *limix.qtl.scan* function after subsetting the full genome SNP matrix [[22]](https://paperpile.com/c/xAKEZR/h5aNN) to include all polymorphic SNPs that have a minor allele frequency (MAF) of at least 5% across the population of 645 accessions. For meta-analyses, only those SNP positions are reported for which the significance level exceeds the Bonferroni corrected threshold with respect to the total number of included SNP markers (1,813,837).

##### Comparison with existing tools

To obtain DMR sets for comparison of MethylScore with DSS [[23]](https://paperpile.com/c/xAKEZR/Qvb7) and metilene [[24]](https://paperpile.com/c/xAKEZR/RnzY), per-context methylation information was extracted and summarized per genotype using MethylDackel extract from alignments of Col-0 wildtype and cmt3 mutant samples (data from [[11]](https://paperpile.com/c/xAKEZR/TUbmE)).

Obtained bedGraph data was first reformatted to compatible input file formats expected by the respective tool and parameters to identify DMRs (for each methylation context separately) were set as follows:

DSS (v2.42.0):

DSS::callDMR(

dmlTest,

delta=0.2,

minCG=3,

p.threshold=0.05

)

metilene (v0.2.8):

metilene \\

-t 8 \\

-m 3 \\

-d 20 \\

-G 2000 #(only set for CHH context to limit memory usage)

Resulting DMRs from metilene were post-hoc filtered to only include regions with associated Bonferroni corrected p-values < 0.05.

##### Data visualization

Figures were generated using *R* 4.1.2 [[20]](https://paperpile.com/c/xAKEZR/lORcQ) and *Bioconductor* 3.14 [[25,26]](https://paperpile.com/c/xAKEZR/LhXVu+yXelM). Heatmaps of methylation rates were generated using the *ComplexHeatmap* library (v2.10.0 [[27]](https://paperpile.com/c/xAKEZR/UyTby). Principal components were computed using the *pcaMethods* library (v1.86.0 [[28]](https://paperpile.com/c/xAKEZR/9kyVu) and visualized with *ggplot2* (v3.3.5 [[29]](https://paperpile.com/c/xAKEZR/8VP7Y) and the *ggrepel* library (v0.9.1 [[30]](https://paperpile.com/c/xAKEZR/u6Cy1). SNP linkage was calculated using the *snpStats* library (v1.44.0) and genomic locations were illustrated using the *gggenes* library (v0.4.1 [[31]](https://paperpile.com/c/xAKEZR/vUban). Genome wide representations of SNP associations were drawn using *ggplot2* (v3.3.5 [[29]](https://paperpile.com/c/xAKEZR/8VP7Y) and the *ggforce* (v0.3.3 [[32]](https://paperpile.com/c/xAKEZR/wwsYe) library for visual annotation. Slopegraphs and Bootstrap Estimation plots were generated using the *dabestr* library (v0.3.0 [[33]](https://paperpile.com/c/xAKEZR/54tyH). Venn diagrams were generated using the *seqsetvis* library (v1.16.0 [[34]](https://paperpile.com/c/xAKEZR/5ISn)).

## Supplementary Figures

##### Supplementary Figure 1

#####
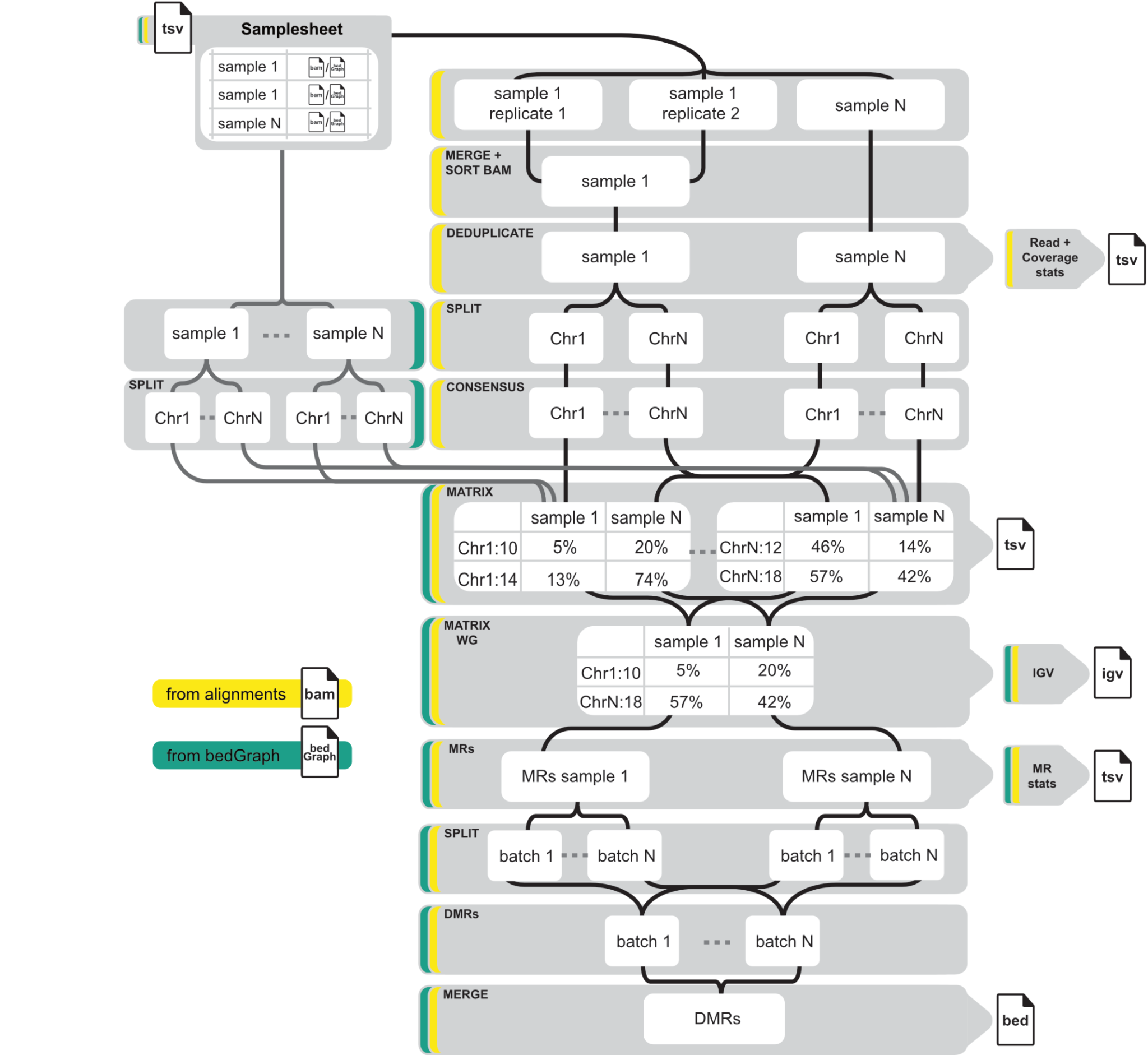


**Supplementary Figure 1. Schematic overview of the MethylScore pipeline.**

Schematic overview of processing steps (grey). Different workflow entry points based on the type of input data are indicated as colored borders. The workflow can be started from genomic alignments (*BAM* format; yellow) or tabular single-cytosine methylation information (*bedGraph* format; green). Chr: chromosome; DMR: differentially methylated region; MR: methylated region.

##### Supplementary Figure 2


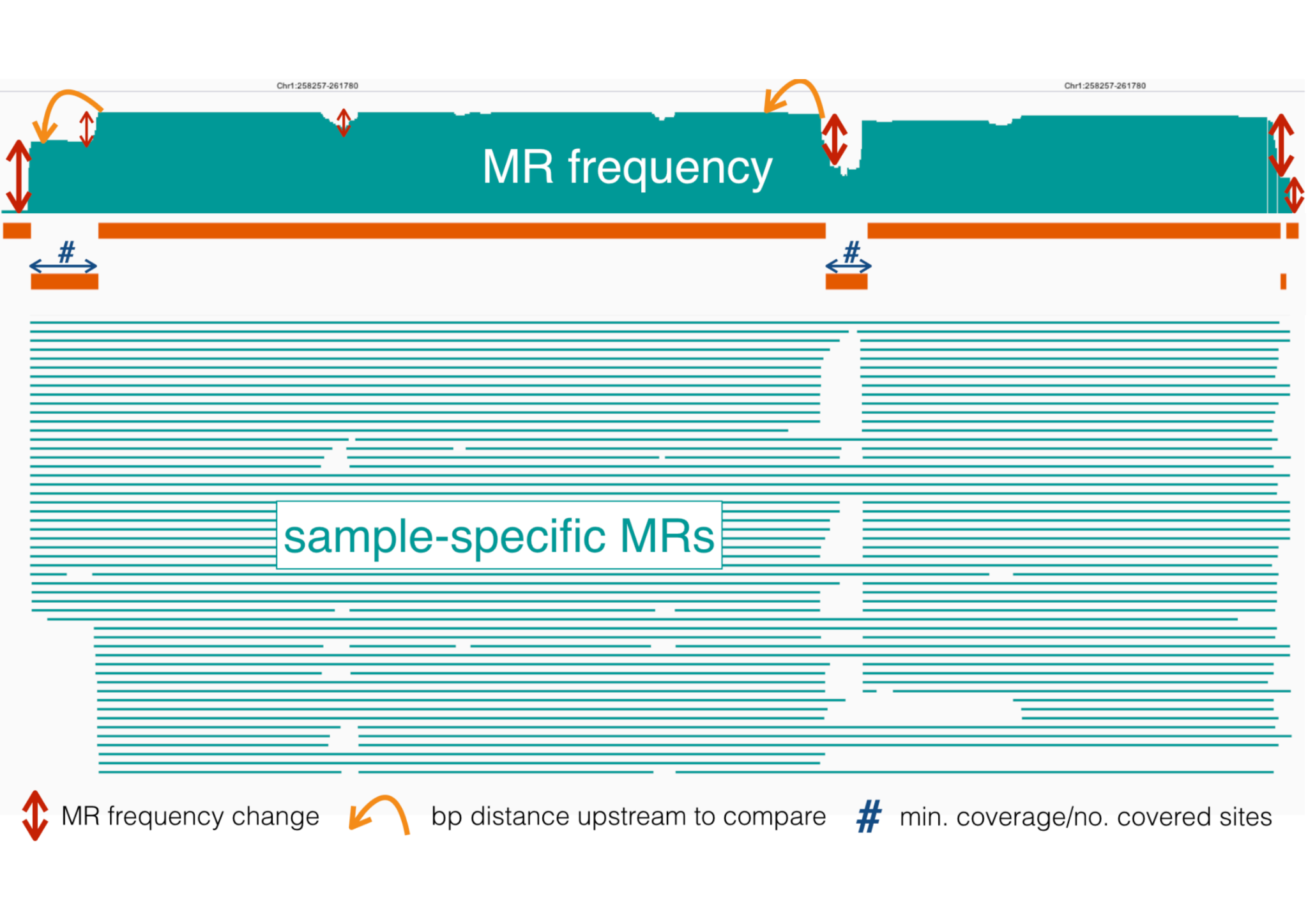


**Supplementary Figure 2: MR-based strategy to determine candidate DMRs.**

The top panel shows a methylation region (MR) frequency bar plot along the genome, summarizing the counts of all sample-specific MRs (the MRs are displayed as horizontal petrol-colored lines in the middle). Three parameters specify the borders of candidate differentially methylated regions (DMRs; orange horizontal bars): 1) the degree of MR frequency changes along the genome (marked by red vertical arrows), 2) the upstream distance to which MR frequency is compared to (marked by orange-colored bent arrows), and 3) a minimum number and coverage of cytosines (blue arrows indicating the length of candidate DMRs).

##### Supplementary Figure 3


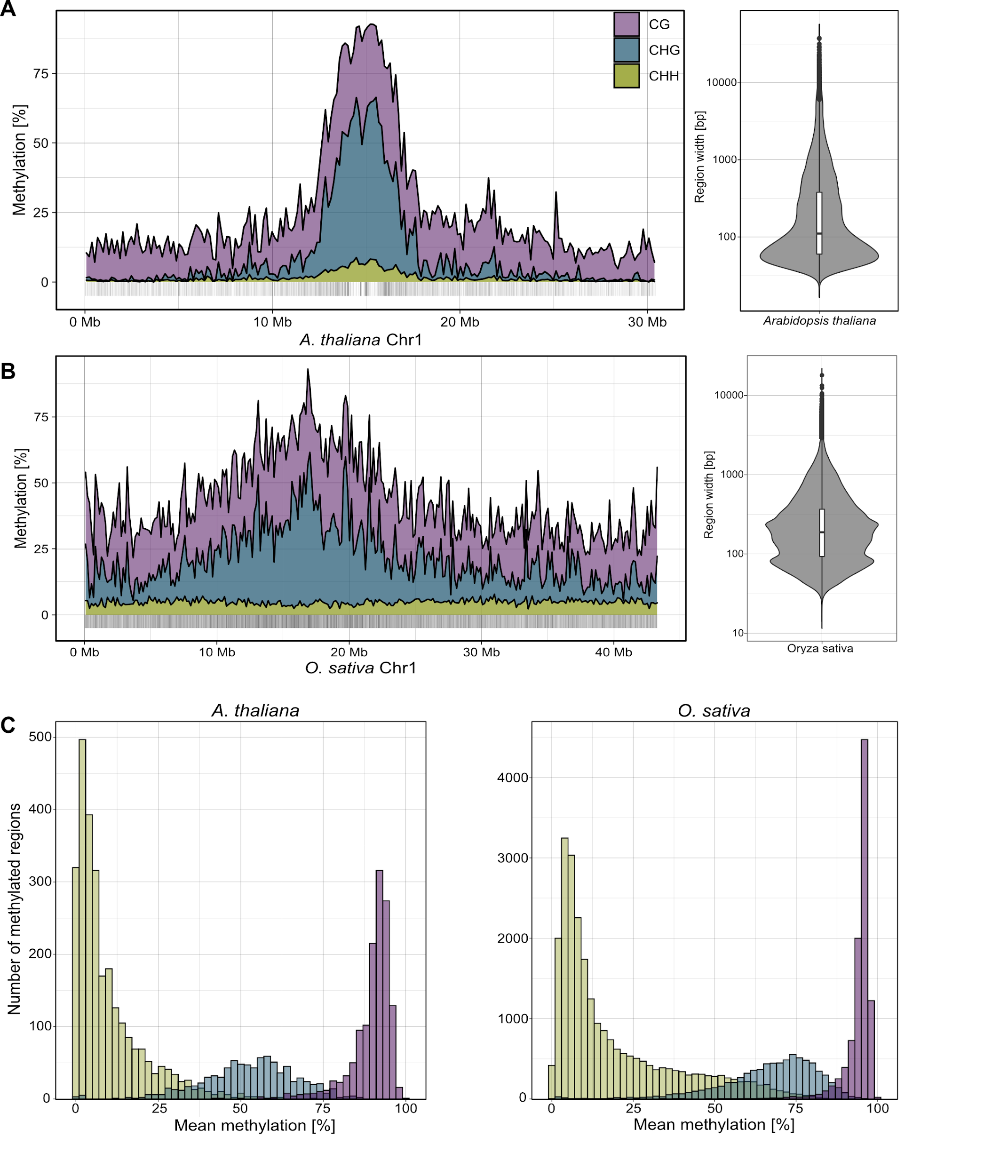


**Supplementary Figure 3: Genomic distribution of DNA methylation and methylated regions (MRs).**

Line plots (left) show the distribution of average DNA methylation in 150 kb bins along single chromosomes of *A. thaliana* (**A;** data from [[18]](https://paperpile.com/c/xAKEZR/KVnQV) and *O. sativa* (**B;** data from [[12]](https://paperpile.com/c/xAKEZR/jV4Wq)*.* MRs segmented by MethylScore are indicated as vertical bars along the *x*-axis, with corresponding length distributions shown as violin plots (right).

Histograms show distribution of context-specific methylation rates within segmented MRs (**C**).

##### Supplementary Figure 4

**
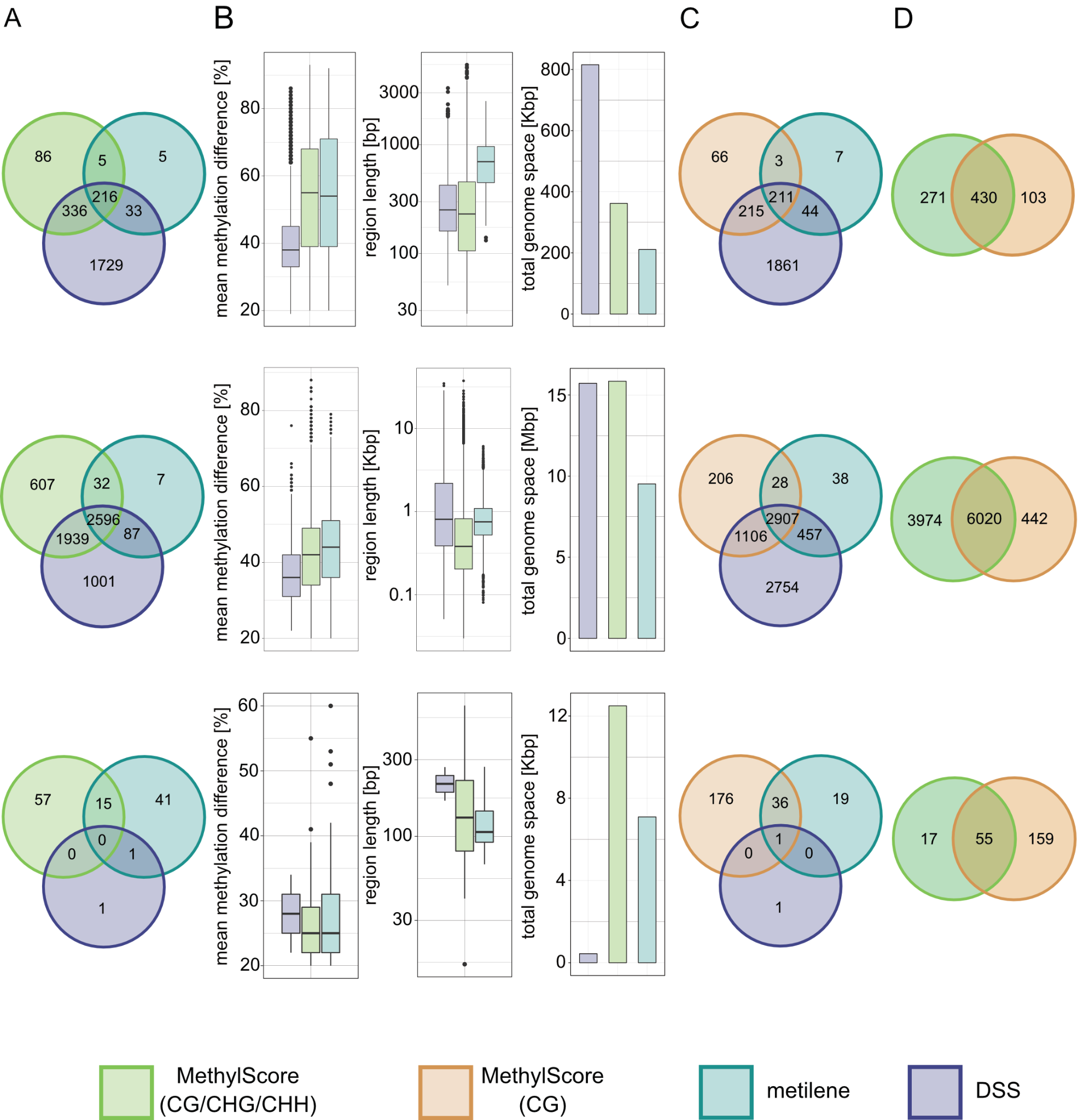
**

**Supplementary Figure 4: Concordance among regions identified as differentially methylated by different DMR callers.**

In all cases, differential methylation was called between *A. thaliana* wild-type and *cmt3* (data from [[11]](https://paperpile.com/c/xAKEZR/TUbmE)). CG, CHG and CHH contexts are shown in top, middle and bottom row, respectively. Three-way Venn diagrams in (**A**) and (**C**) show the number of intersecting regions identified by MethylScore (using context-specific Hidden Markov Model (**A**), or a CG-only model (**C**)), metilene [[24]](https://paperpile.com/c/xAKEZR/RnzY), and DSS [[23]](https://paperpile.com/c/xAKEZR/Qvb7). Two-way Venn diagrams (**D**) show overlap between DMRs identified from context-specific and CG-only models. **B**) Boxplots show the mean methylation difference (left), length distribution (centre) and total covered genome space (right) of regions identified as differentially methylated by different tools.

##### Supplementary Figure 5


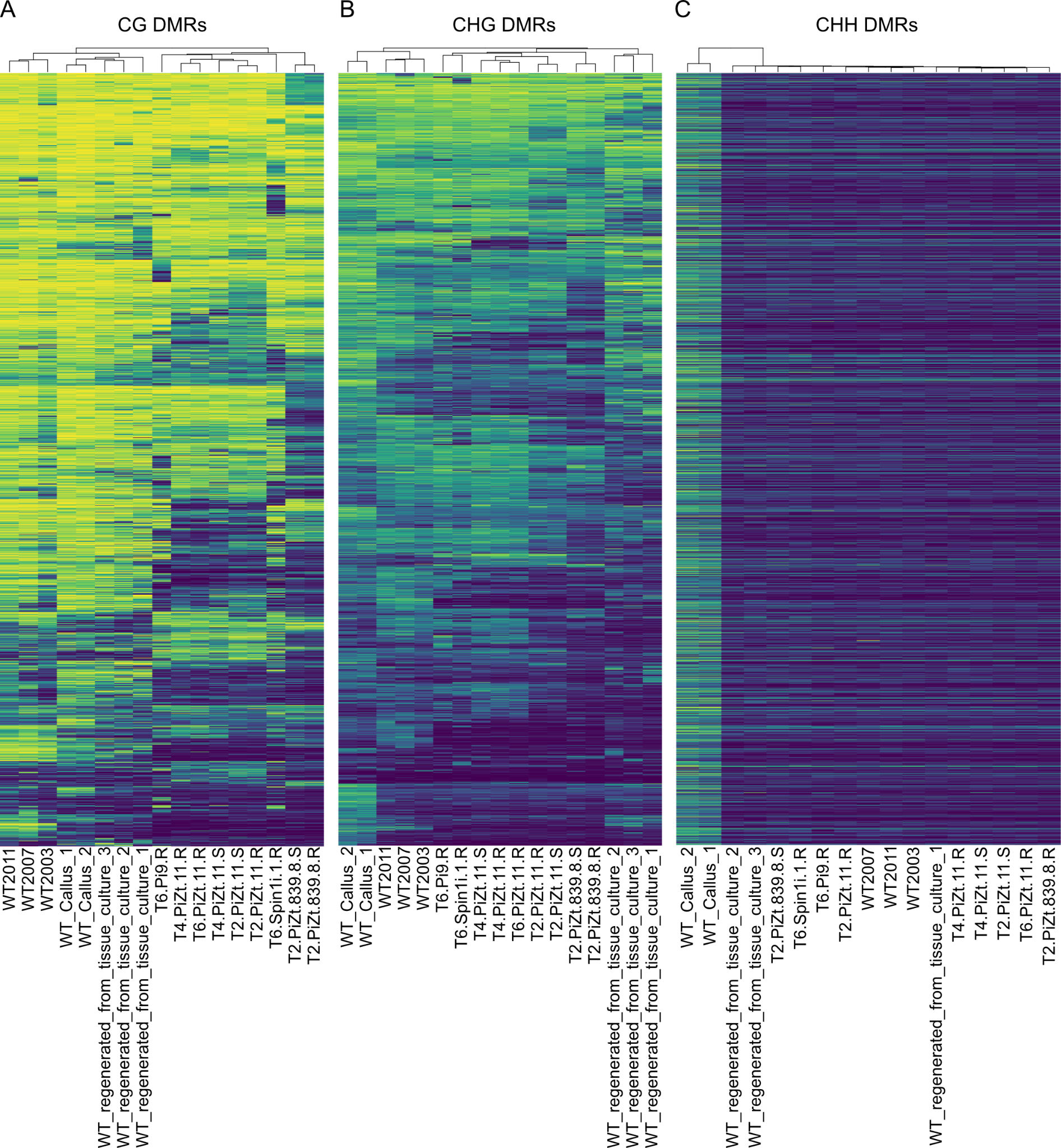


**Supplementary Figure 5: Differential methylation in rice regenerants.**

Heatmaps show methylation rate averages in regions identified as differentially methylated in CG, CHG, or CHH methylation contexts (from left to right) in WGBS data from different rice regenerants, callus tissue, and non-regenerated controls. Original data from [[12]](https://paperpile.com/c/xAKEZR/jV4Wq), GEOaccession GSE42410.

##### Supplementary Figure 6


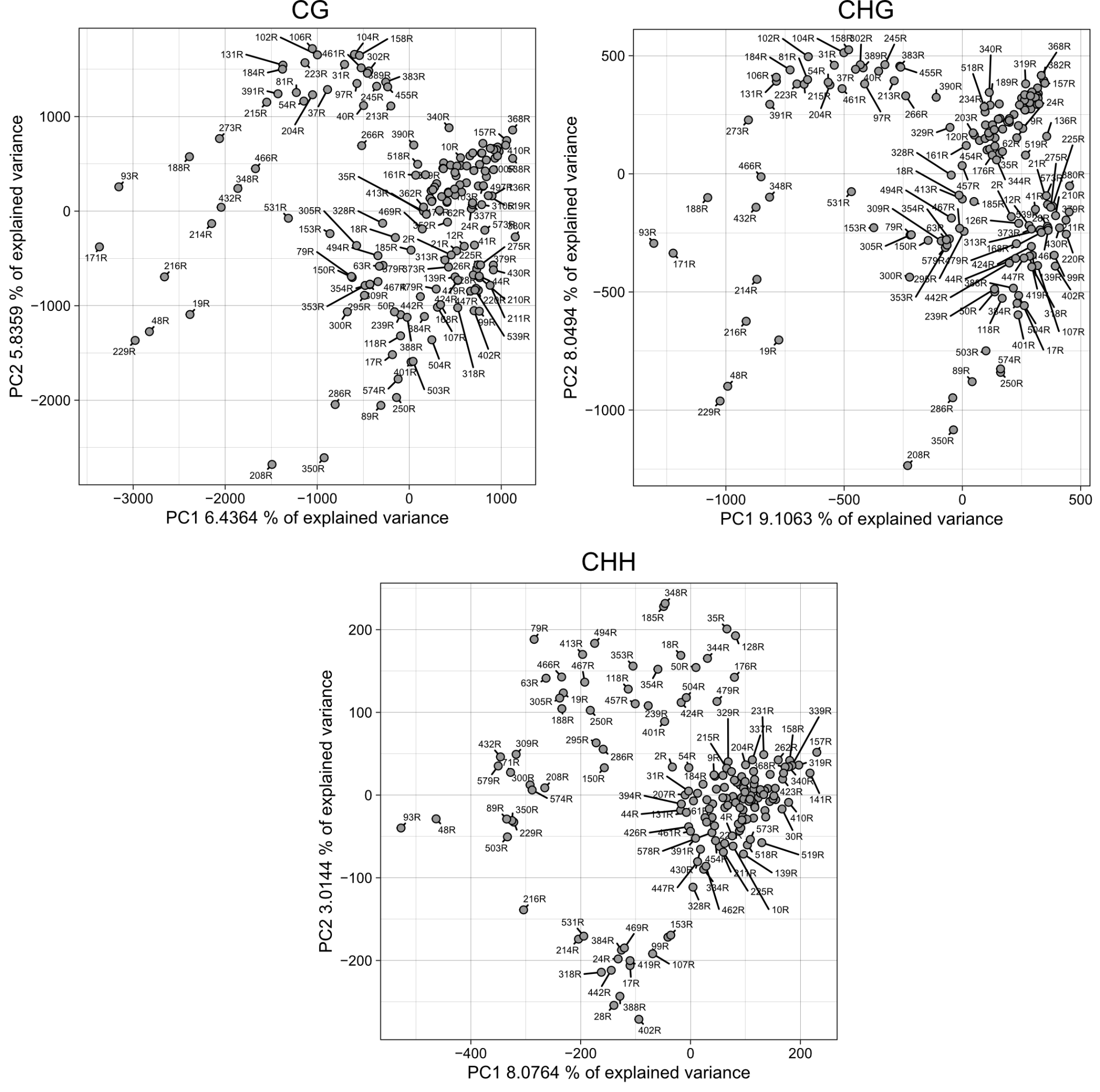


**Supplementary Figure 6: DMR identification across epigenetic recombinant inbred lines (epiRILs).**

Principal component analysis (PCA) of mean methylation rates within 46,323 CG-, 8,821 CHG- and 4,121 CHH-DMRs identified by MethylScore across a population of 169 epiRILs.

##### Supplementary Figure 7


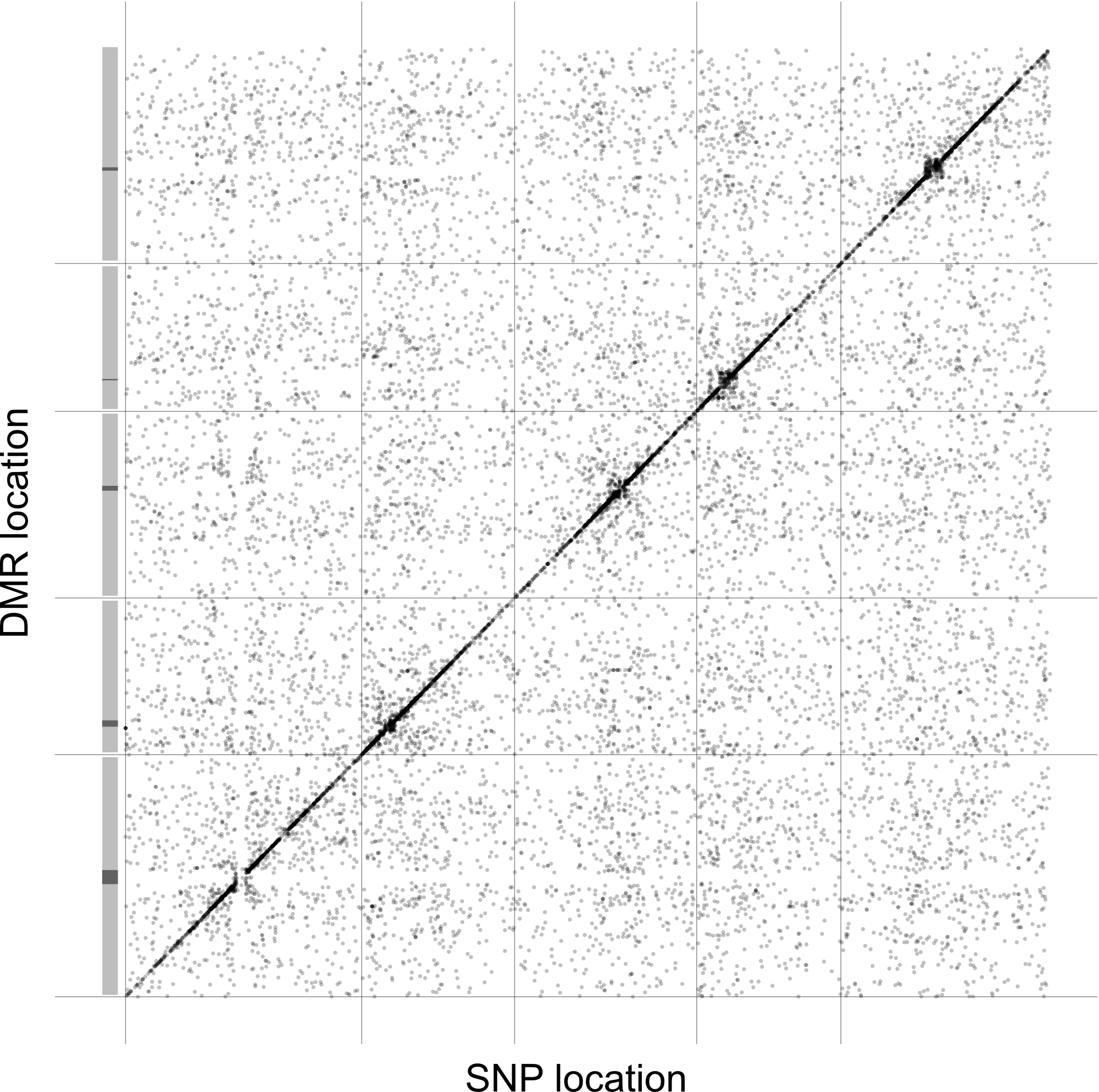


**Supplementary Figure 7: GWA analysis on region-level methylation rate averages in CG.**

Supplementary Figure 8

**Supplementary Figure 8: Resource requirements of individual pipeline components.**
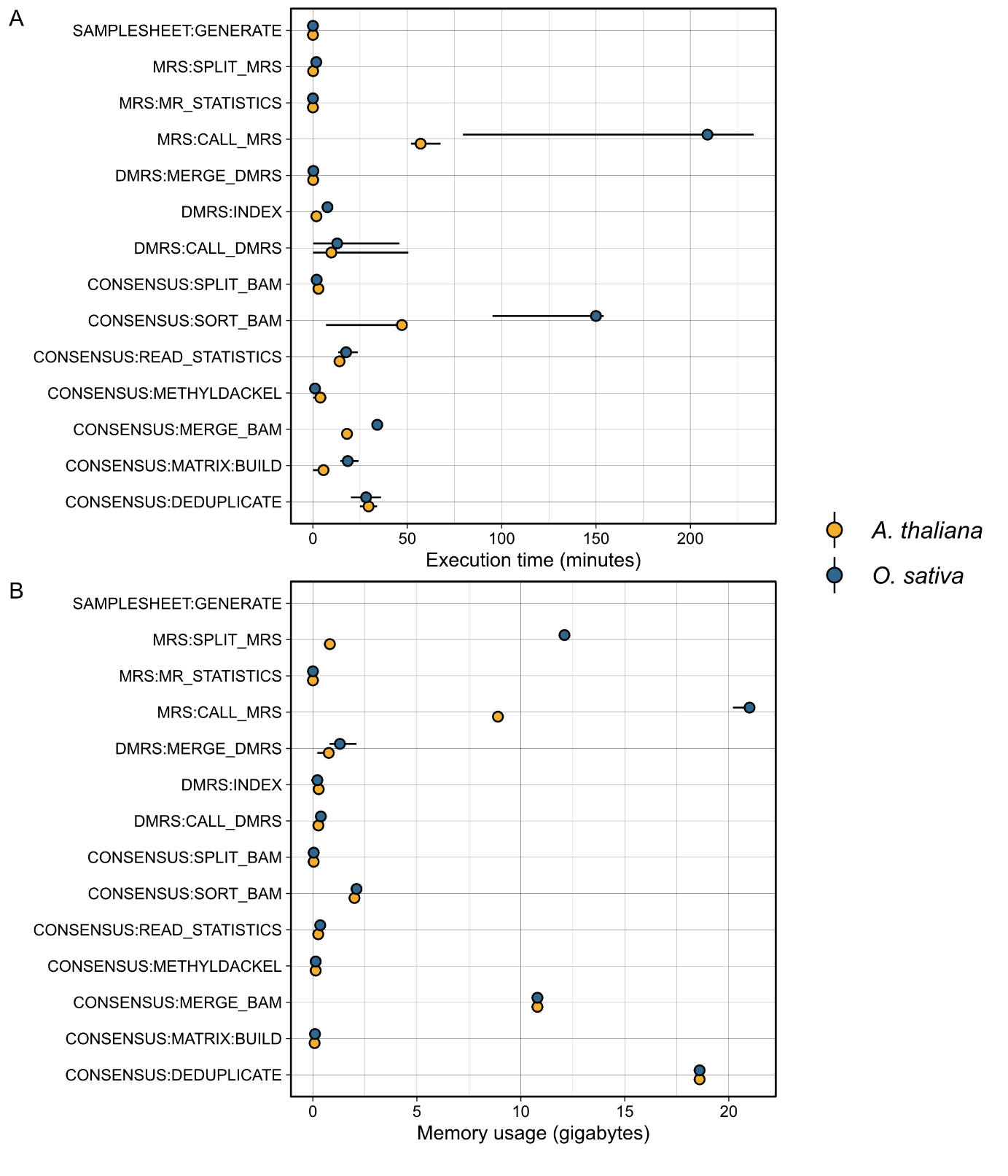


Supplementary Figure 9


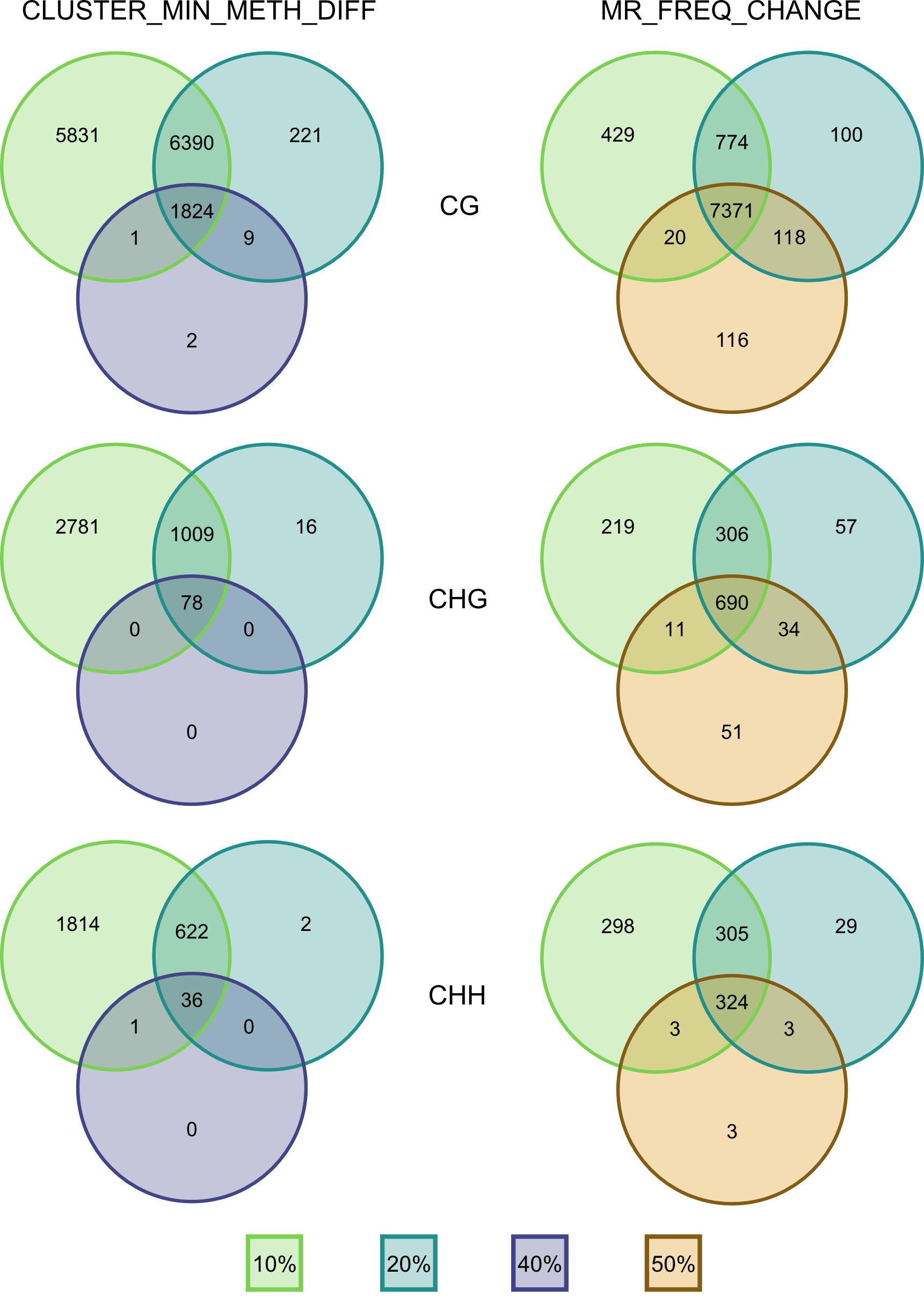

**Supplementary Figure 9: Effect of parameter changes on the number of identified regions.**

Venn diagrams show the concordance of DMR calls (data from [[35]](https://paperpile.com/c/xAKEZR/vcnx)) when applying different thresholds for the mean methylation difference between sample clusters (left: CLUSTER_MIN_METH_DIFF) and for the change of MR frequency along the genome (right: MR_FREQ_CHANGE)**.**

## Supplementary references

1. [Li H, Handsaker B, Wysoker A, Fennell T, Ruan J, Homer N, et al. The Sequence Alignment/Map format and SAMtools. Bioinformatics. 2009;25: 2078–2079.](http://paperpile.com/b/xAKEZR/artBC)

2. [Picard toolkit. Broad Institute, GitHub repository. Broad Institute; 2019. Available:](http://paperpile.com/b/xAKEZR/zOhN4) <https://broadinstitute.github.io/picard/>

3. [Molaro A, Hodges E, Fang F, Song Q, McCombie WR, Hannon GJ, et al. Sperm methylation profiles reveal features of epigenetic inheritance and evolution in primates. Cell. 2011;146: 1029–1041.](http://paperpile.com/b/xAKEZR/6IuHF)

4. [MacQueen J. Some methods for classification and analysis of multivariate observations. Proceedings of the Fifth Berkeley Symposium on Mathematical Statistics and Probability, Volume 1: Statistics. Berkeley, CA: University of California Press; 1967. pp. 281–298.](http://paperpile.com/b/xAKEZR/hw7vk)

5. [Hagmann J, Becker C, Müller J, Stegle O, Meyer RC, Wang G, et al. Century-scale methylome stability in a recently diverged Arabidopsis thaliana lineage. PLoS Genet. 2015;11: e1004920.](http://paperpile.com/b/xAKEZR/CzcUP)

6. [Storey JD, Tibshirani R. Statistical significance for genomewide studies. Proc Natl Acad Sci U S A. 2003;100: 9440–9445.](http://paperpile.com/b/xAKEZR/fq56L)

7. [Becker C, Hagmann J, Müller J, Koenig D, Stegle O, Borgwardt K, et al. Spontaneous epigenetic variation in the Arabidopsis thaliana methylome. Nature. 2011;480: 245–249.](http://paperpile.com/b/xAKEZR/AZd3h)

8. [Di Tommaso P, Chatzou M, Floden EW, Barja PP, Palumbo E, Notredame C. Nextflow enables reproducible computational workflows. Nat Biotechnol. 2017;35: 316–319.](http://paperpile.com/b/xAKEZR/ogr06)

9. [Wibowo A, Becker C, Marconi G, Durr J, Price J, Hagmann J, et al. Hyperosmotic stress memory in Arabidopsis is mediated by distinct epigenetically labile sites in the genome and is restricted in the male germline by DNA glycosylase activity. eLife Sciences. 2016;5: e13546.](http://paperpile.com/b/xAKEZR/uey7n)

10. [Tedeschi F, Rizzo P, Huong BTM, Czihal A, Rutten T, Altschmied L, et al. EFFECTOR OF TRANSCRIPTION factors are novel plant-specific regulators associated with genomic DNA methylation in Arabidopsis. New Phytol. 2019;221: 261–278.](http://paperpile.com/b/xAKEZR/niFZ2)

11. [Ning Y-Q, Liu N, Lan K-K, Su Y-N, Li L, Chen S, et al. DREAM complex suppresses DNA methylation maintenance genes and precludes DNA hypermethylation. Nat Plants. 2020;6: 942–956.](http://paperpile.com/b/xAKEZR/TUbmE)

12. [Stroud H, Ding B, Simon SA, Feng S, Bellizzi M, Pellegrini M, et al. Plants regenerated from tissue culture contain stable epigenome changes in rice. Elife. 2013;2: e00354.](http://paperpile.com/b/xAKEZR/jV4Wq)

13. [Zhang Y, Jang H, Xiao R, Kakoulidou I, Piecyk RS, Johannes F, et al. Heterochromatin is a quantitative trait associated with spontaneous epiallele formation. Nat Commun. 2021;12: 1–13.](http://paperpile.com/b/xAKEZR/C2opY)

14. [Lamesch P, Berardini TZ, Li D, Swarbreck D, Wilks C, Sasidharan R, et al. The Arabidopsis Information Resource (TAIR): improved gene annotation and new tools. Nucleic Acids Res. 2012;40: D1202–10.](http://paperpile.com/b/xAKEZR/RUBMW)

15. [Kawahara Y, de la Bastide M, Hamilton JP, Kanamori H, McCombie WR, Ouyang S, et al. Improvement of the Oryza sativa Nipponbare reference genome using next generation sequence and optical map data. Rice . 2013;6: 4.](http://paperpile.com/b/xAKEZR/961Aa)

16. [Ewels P, Hüther P, Hammarén R, mashehu, Peltzer A, Bot N-C, et al. nf-core/methylseq: nf-core/methylseq version 1.6.1 [Nauseous Serpent]. 2021. doi:](http://paperpile.com/b/xAKEZR/3LHRw)[10.5281/zenodo.4744708](http://dx.doi.org/10.5281/zenodo.4744708)

17. [Krueger F, Andrews SR. Bismark: a flexible aligner and methylation caller for Bisulfite-Seq applications. Bioinformatics. 2011;27: 1571–1572.](http://paperpile.com/b/xAKEZR/LTz5u)

18. [Kawakatsu T, Huang S-SC, Jupe F, Sasaki E, Schmitz RJ, Urich MA, et al. Epigenomic Diversity in a Global Collection of Arabidopsis thaliana Accessions. Cell. 2016;166: 492–505.](http://paperpile.com/b/xAKEZR/KVnQV)

19. [Lee S, Cook D, Lawrence M. plyranges: a grammar of genomic data transformation. Genome Biol. 2019;20: 4.](http://paperpile.com/b/xAKEZR/fSic8)

20. [R Core Team. R: A Language and Environment for Statistical Computing. Vienna, Austria: R Foundation for Statistical Computing; 2021. Available:](http://paperpile.com/b/xAKEZR/lORcQ) [https://www.R-project.org/](https://www.r-project.org/)

21. [Lippert C, Casale FP, Rakitsch B, Stegle O. LIMIX: genetic analysis of multiple traits. bioRxiv. 2014. p. 003905. doi:](http://paperpile.com/b/xAKEZR/gaLxL)[10.1101/003905](http://dx.doi.org/10.1101/003905)

22. [1001 Genomes Consortium. 1,135 Genomes Reveal the Global Pattern of Polymorphism in Arabidopsis thaliana. Cell. 2016;166: 481–491.](http://paperpile.com/b/xAKEZR/h5aNN)

23. [Feng H, Conneely KN, Wu H. A Bayesian hierarchical model to detect differentially methylated loci from single nucleotide resolution sequencing data. Nucleic Acids Res. 2014;42: e69.](http://paperpile.com/b/xAKEZR/Qvb7)

24. [Jühling F, Kretzmer H, Bernhart SH, Otto C, Stadler PF, Hoffmann S. metilene: fast and sensitive calling of differentially methylated regions from bisulfite sequencing data. Genome Res. 2016;26: 256–262.](http://paperpile.com/b/xAKEZR/RnzY)

25. [Gentleman RC, Carey VJ, Bates DM, Bolstad B, Dettling M, Dudoit S, et al. Bioconductor: open software development for computational biology and bioinformatics. Genome Biol. 2004;5: R80.](http://paperpile.com/b/xAKEZR/LhXVu)

26. [Huber W, Carey VJ, Gentleman R, Anders S, Carlson M, Carvalho BS, et al. Orchestrating high-throughput genomic analysis with Bioconductor. Nat Methods. 2015;12: 115–121.](http://paperpile.com/b/xAKEZR/yXelM)

27. [Gu Z, Eils R, Schlesner M. Complex heatmaps reveal patterns and correlations in multidimensional genomic data. Bioinformatics. 2016;32: 2847–2849.](http://paperpile.com/b/xAKEZR/UyTby)

28. [Stacklies W, Redestig H, Scholz M, Walther D, Selbig J. pcaMethods—a bioconductor package providing PCA methods for incomplete data. Bioinformatics. 2007;23: 1164–1167.](http://paperpile.com/b/xAKEZR/9kyVu)

29. [Wickham H. ggplot2: Elegant Graphics for Data Analysis. Springer, New York, NY; 2009.](http://paperpile.com/b/xAKEZR/8VP7Y)

30. [Slowikowski K. ggrepel: Repel overlapping text labels away from each other. Github; Available:](http://paperpile.com/b/xAKEZR/u6Cy1) <https://github.com/slowkow/ggrepel>

31. [Wilkins D. gggenes: ➡️️➡️️⬅️️➡️️ Draw gene arrow maps in ggplot2. Github; Available:](http://paperpile.com/b/xAKEZR/vUban) <https://github.com/wilkox/gggenes>

32. [Pedersen TL. ggforce: Accelerating ggplot2. Github; Available:](http://paperpile.com/b/xAKEZR/wwsYe) <https://github.com/thomasp85/ggforce>

33. [Ho J, Tumkaya T, Aryal S, Choi H, Claridge-Chang A. Moving beyond P values: data analysis with estimation graphics. Nat Methods. 2019;16: 565–566.](http://paperpile.com/b/xAKEZR/54tyH)

34. [Boyd JR. seqsetvis: Set Based Visualizations for Next-Gen Sequencing Data. 2022.](http://paperpile.com/b/xAKEZR/5ISn)

35. [Wibowo A, Becker C, Durr J, Price J, Spaepen S, Hilton S, et al. Partial maintenance of organ-specific epigenetic marks during plant asexual reproduction leads to heritable phenotypic variation. Proc Natl Acad Sci U S A. 2018;115: E9145–E9152.](http://paperpile.com/b/xAKEZR/vcnx)
